# Supplementary material for: An open-source framework for large-scale, flexible evaluation of biomedical text mining systems
Source: J Biomed Discov Collab. 2008 Jan 29;3:1. doi: 10.1186/1747-5333-3-1 (PMC2276192; doi:10.1186/1747-5333-3-1)
Supplement: Additional file 1 — Evaluation platform source code. The additional file contains the software evaluation framework discussed in this paper. The most current release can be downloaded from SourceForge.net [1]. See the accompanying README file for instructions on installation and use. Also included in the distribution is a collection of UIMA wrappers for some commonly used BioNLP tools and annotated corpora. [file 1747-5333-3-1-S1.zip › bionlp_uima_repository-1.0.1/docs/api/src-html/edu/uchsc/ccp/util/external/penn/PennBioTaggerEISystem.html]

```
001    package edu.uchsc.ccp.util.external.penn;
002    
003    import java.io.IOException;
004    import java.util.ArrayList;
005    import java.util.List;
006    
007    import edu.uchsc.ccp.util.nlp.annotation.Annotator;
008    import edu.uchsc.ccp.util.nlp.annotation.TextAnnotation;
009    import edu.uchsc.ccp.util.nlp.ei.EISystem;
010    import edu.uchsc.ccp.util.nlp.ei.xml.XMLEntityExtractor;
011    import edu.uchsc.ccp.util.nlp.mention.ClassMentionTypes;
012    import edu.uchsc.ccp.util.nlp.mention.ComplexSlotMentionTypes;
013    import edu.upenn.cis.taggers.LoadModelException;
014    import edu.upenn.cis.taggers.Tagger;
015    import edu.upenn.cis.taggers.gene.GeneTagger;
016    import edu.upenn.cis.taggers.malignancy.MalignancyTagger;
017    import edu.upenn.cis.taggers.variation.VariationTagger;
018    
019    public class PennBioTaggerEISystem implements EISystem {
020    private final boolean DEBUG = false;
021    
022        public static final String TAGGER_TYPE_GENE = "gene";
023    
024        public static final String TAGGER_TYPE_VARIATION = "variation";
025    
026        public static final String TAGGER_TYPE_MALIGNANCY = "malignancy";
027    
028        private Tagger tagger;
029    
030        private final String[] geneTags = {"gene"};
031        private final String[] geneClassMentionTypes = {ClassMentionTypes.PROTEIN};
032        private final String[] varTags = {"type","state-original","state-altered","location","event"};
033        private final String[] varClassMentionTypes = {ClassMentionTypes.VARIATION_TYPE,ComplexSlotMentionTypes.SUBSTITUTION_WILDTYPE_ELEMENT,ComplexSlotMentionTypes.SUBSTITUTION_MUTANT_ELEMENT,ClassMentionTypes.BIOLOGICAL_SEQUENCE_POSITION,ClassMentionTypes.VARIATION_EVENT};
034        private final String[] malTags = {"malignancy-type"};
035        private final String[] malClassMentionTypes = {ClassMentionTypes.MALIGNANCY};
036    
037        private String[] classMentionTypesStr;
038        private String[] tagsStr;
039        
040        private final Annotator annotator = new Annotator(new Integer(21),"","PennBioTagger","UPenn");
041    
042        /**
043         * Initialize a new tagger. Tagger can either be for genes, variations, or malignancies.
044         * 
045         * @param taggerType
046         * @param entityModelFile
047         * @param tokenizerModelFile
048         */
049        public PennBioTaggerEISystem(String taggerType, String entityModelFile, String tokenizerModelFile) {
050            try {
051                if (taggerType.equals(TAGGER_TYPE_GENE)) {
052                    tagger = new GeneTagger(entityModelFile, tokenizerModelFile);
053                    tagsStr = geneTags;
054                    classMentionTypesStr = geneClassMentionTypes;
055                } else if (taggerType.equals(TAGGER_TYPE_VARIATION)) {
056                    tagger = new VariationTagger(entityModelFile, tokenizerModelFile);
057                    tagsStr = varTags;
058                    classMentionTypesStr = varClassMentionTypes;
059                } else if (taggerType.equals(TAGGER_TYPE_MALIGNANCY)) {
060                    tagger = new MalignancyTagger(entityModelFile, tokenizerModelFile);
061                    tagsStr = malTags;
062                    classMentionTypesStr = malClassMentionTypes;
063                } else {
064                    System.err.println("Invalid tagger type parameter. Valid choices are: " + TAGGER_TYPE_GENE + ", " + TAGGER_TYPE_VARIATION
065                            + ", or " + TAGGER_TYPE_MALIGNANCY);
066                    tagger = null;
067                    taggerType = null;
068                    classMentionTypesStr = null;
069                }
070            } catch (LoadModelException e) {
071                e.printStackTrace();
072            }
073        }
074    
075        /**
076         * Find entities in a particular String of text.
077         */
078        public List<TextAnnotation> getEntitiesFromString(String inputText, String inputDocumentID) {
079    
080            String taggedText = null;
081            try {
082                taggedText = tagger.tag(inputText);
083            } catch (IOException ioe) {
084                System.err.println("Tagger failed while tagging text...");
085                ioe.printStackTrace();
086            }
087            
088            if (DEBUG) {
089                System.out.println("DEBUG PENNBIOTAGGER - TAGGED TEXT: " + taggedText);
090            }
091            
092            List<TextAnnotation> entities = new ArrayList<TextAnnotation>();
093            for (int i = 0; i < tagsStr.length; i++) {
094                List<TextAnnotation> some_entities = XMLEntityExtractor.parseEntitiesFromSparseXML(taggedText, inputText, tagsStr[i], inputDocumentID, classMentionTypesStr[i], annotator);
095                entities.addAll(some_entities);
096            }
097            
098            return entities;
099        }
100    
101    }
```
